# Spectrum and management of breast cancer patients with variant of uncertain significance mutations at a tertiary care centre in North India

**DOI:** 10.3332/ecancer.2022.1434

**Published:** 2022-08-01

**Authors:** Abhenil Mittal, S V S Deo, Ajay Gogia, Atul Batra, Akash Kumar, Sandeep Bhoriwal, Koushik Sinha Deb, Ekta Dhamija, V L Ramprasad, Olufunmilayo Olopade, Raja Pramanik

**Affiliations:** 1 Department of Medical Oncology, All India Institute of Medical Sciences, New Delhi 110029, India; 2 Department of Surgical Oncology, All India Institute of Medical Sciences, New Delhi 110029, India; 3 Department of Medical Oncology, National Cancer Institute, Jhajjar, Haryana 110029, India; 4 Department of Psychiatry, All India Institute of Medical Sciences, New Delhi 110029, India; 5 Department of Radiodiagnosis, All India Institute of Medical Sciences, New Delhi 110029, India; 6 MedGenome Labs Ltd, Bengaluru, Karnataka 560099, India; 7 Center for Clinical Cancer Genetics and Global Health, University of Chicago, Chicago, IL 60637, USA

**Keywords:** variant of uncertain significance, next-generation sequencing, breast cancer, India

## Abstract

**Background:**

The spectrum and significance of Variants of Uncertain Significance (VUS) mutations in breast cancer predisposition genes is poorly defined in the Indian population.

**Methods:**

All new female breast cancer patients from 1 March 2019 to 28 February 2020 were screened. Those providing informed consent and without previous genetic testing were recruited. Multigene panel testing (107 genes) by next-generation sequencing was performed for all patients. Descriptive statistics was used to describe the spectrum of VUS mutations.

**Results:**

Out of 236 patients recruited in the study, a VUS was detected in 89 patients (37.71%). VUS pathogenic ratio was 2.02. A total of 121 different VUS mutations in 40 different genes were detected. Fourteen patients (15.7%) had a VUS in high penetrance genes and 36 VUS mutations (29.8%) were detected in one of the genes involved in homologous recombination repair pathway. No therapeutic interventions were done based on VUS.

**Conclusions:**

In this large prospective study of genetic determinants of breast cancer from India, a high prevalence of VUS (37.71%) was detected with 15.7% patients having a VUS in high penetrance genes. More evidence needs to be generated from larger multicentric studies to better understand the implications of these genetic variants and enable their reclassification.

## Introduction

Breast cancer is the most prevalent cancer worldwide and is also the most common cancer among Indian women. It also constitutes the major cause of mortality worldwide and in India [1, 2]. Approximately 10%–15% of breast cancers are hereditary and mutations in BRCA 1/2 account for half of the hereditary cancer burden (4%–6% of unselected breast cancer population) [3–7]. With the decreasing cost of genetic testing and increasing availability, the paradigm has shifted towards selecting patients who should not be offered testing, rather than selecting patients for germline testing [8]. Previous studies from large US based institutions showed that National Comprehensive Cancer Center Criteria (NCCN) were relatively insensitive to select patients for genetic testing and up to 50% patients with pathogenic/likely pathogenic mutations may be missed if only NCCN criteria are used to select patients. Work from our centre also showed that sensitivity of these criteria was suboptimal when applied to an Indian breast cancer population and we suggested relaxing the age cut off in India to include all women with breast cancer up to 60 years of age to achieve optimal sensitivity, however, this data does need validation in larger studies [9–12].

The optimum method of genetic testing still remains a matter of debate and depends on clinical setting, cost and availability [13]. However, in recent years, multi-gene panels have gained popularity and have become the standard of care for hereditary breast cancer at most institutions [14–16]. Advantages include saving both money and time, detection of many uncommon high penetrance genes and moderate penetrance genes for which evidence base is now accumulating and availability of comprehensive genomic information which can help in counselling patients and their families better and make informed therapeutic decisions [17, 18]. Multi-gene panels do come with their own set of issues including detection of pathogenic variants in genes for which management strategies are not well defined as yet and high rate of variants of uncertain significance (VUS).

VUS are defined by both the American College of Medical Genetics (ACMG) and International Agency for Research on Cancer as variants with scarce clinical information due to lack of population level evidence, scarce functional evidence, different evaluation and interpretation by clinicians and researchers and being usually found in non-coding regions as mis-sense or synonymous substitutions [13, 19, 20]. Unfortunately, VUS are the most frequently detected variants by next-generation sequencing (NGS), especially in high-risk genes like BRCA1/2, accounting for >40% of all variants detected with pathogenic/likely pathogenic variants accounting for <20% [19, 21]. The ever-growing accumulation of genetic data generates larger and larger percentages of VUS, and this is especially true of oncological diseases, for which large gene-panel sequencing is often required [22, 23]. It is expected that the frequency of VUS will decrease as more data accumulates from various races and ethnic backgrounds.

Majority of data on VUS in various international databases are derived from studies in western population [24, 25]. Data regarding VUS from India has been hardly reported in previous studies reflecting an urgent need for incorporating data from Indian patients in these databases [26, 27]. Therefore, in this study, we report on the spectrum of VUS mutations in consecutive breast cancer patients treated at an Indian tertiary care centre.

## Methods

We screened all new breast cancer patients above the age of 18 years registered at our Institute’s breast cancer clinic over a 1-year period for participation in the study. Patients who had undergone previous germline genetic testing or who did not consent were excluded. After recording a detailed clinical history in a predesigned proforma, a three-generation pedigree chart was built based on family history. American Joint Committee on Cancer 8th edition was used to stage the patients. All patients were grouped into those qualifying the NCCN 2018 criteria or Mainstreaming Cancer Genetics (MCG) Plus criteria [28] for testing and those who do not using a predesigned checklist (Supplementary Tables 1 and 2). After the patients had been through pre-test genetic counselling by a medical oncologist, a 5 ml peripheral blood draw was made at the first visit and subjected to targeted NGS using the TruSight Cancer Sequencing Panel (Illumina, USA). The panel covered 107 high-risk genes known to be associated with cancer predisposition and all testing was performed at a centralised College of American Pathologists and Clinical Laboratory Improvement Amendments certified laboratory. Reflex multiplex ligation by probe amplification (MLPA method) was done to identify large genomic rearrangements in BRCA1 and 2 genes in cases where NGS did not reveal a pathogenic mutation.

**Figure 1.**
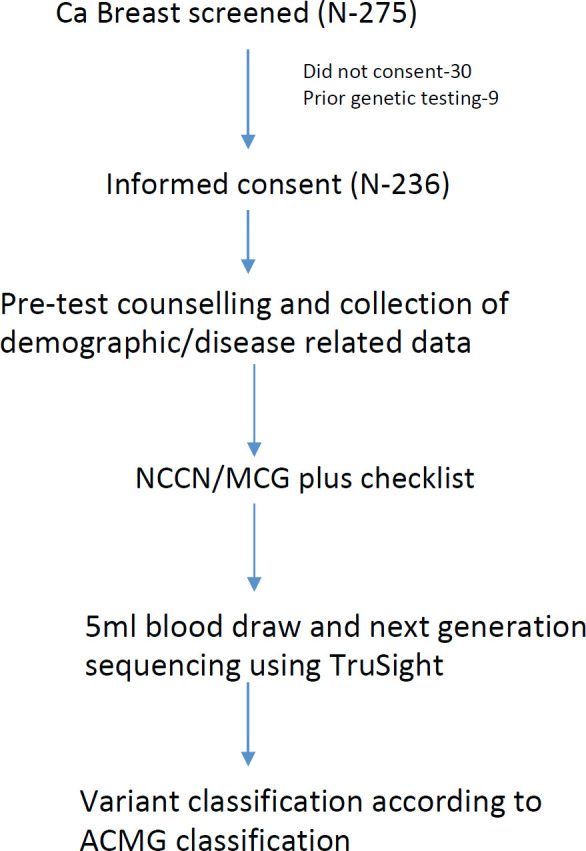
Study schema.

Variants thus obtained by NGS/MLPA were grouped as benign, likely benign, pathogenic, likely pathogenic and VUS according to the ACMG Guidelines (Figure 1). Sanger sequencing was performed to confirm pathogenic/likely pathogenic variants. The study was approved by the Institute Ethics Committee (IEC) and was done according to good clinical practise guidelines as outlined in the Declaration of Helsinki.

### Technical details of NGS and variant interpretation

From 50 ng of input genomic DNA of each patient, NGS libraries were prepared and hybridised to a custom pool of oligonucleotides, targeting genomic regions followed by paired end sequencing of up to 150 bp read lengths. DNA extracted from blood was used to perform targeted gene capture using a custom capture kit. The libraries were sequenced to mean > 80–100× coverage on Illumina sequencing platform. The sequences obtained were aligned to human reference genome (GRCh37/hg19) using the Burrows Wheeler Aligner (BWA) program and analysed using Picard and the Genome Analysis Toolkit (GATK) version 3.6 to identify variants relevant to the clinical indication. The GATK best practices framework was followed for identification of variants in the sample. Gene annotation of the variants was performed using the Variant Effect Predictor (VEP) program against the Ensembl release 87 human gene model. Clinically relevant mutations were annotated using published variants in literature and a set of diseases databases – ClinVar, OMIM, GWAS, HGMD and SwissVar. Common variants were filtered based on allele frequency in 1000 Genome Phase 3, ExAC, EVS, dbSNP147, 1000 Japanese Genome and our internal Indian population database. Non-synonymous variants effect was calculated using multiple algorithms such as PolyPhen-2, Scale Invariant Future Transform (SIFT), Mutation Taster2, Mutation Assessor and Likelihood Ratio Test (LRT). Only non-synonymous and splice site variants found in the hereditary cancer gene panel were used for clinical interpretation. Genetic test results were reported based on the recommendations of ACMG.

## Results

### Baseline characteristics between patients with and without VUS

A total of 236 breast cancer patients were recruited in the study out of 275 that were screened. Nine patients (3.3%) had previous germline testing and 30 patients (10.9%) did not consent.

Out of these 236 patients, a VUS was detected in 89 patients (37.71%). Mean age of this cohort was 46.2 years (18–69 years) and this was similar to patients who did not have a VUS (Table 1). Among patients with VUS, 5 patients (5.6%) had stage I disease, 27 patients (30.3%) had stage II disease and 31 (34.8%) and 26 patients (29.2%) had stage III and metastatic disease, respectively. The stage distribution was similar in patients with and without VUS. Patients with VUS were more often Her2 positive (42.7% versus 27.9%, *p* = 0.019) but significant family history was seen less often (8% versus 22.5%, *p* = 0.026). Rates of triple negative breast cancer (30.3% versus 38%) were similar between patients irrespective of VUS. Other baseline characteristics are detailed in Table 1.

### VUS characteristics

A total of 121 VUS mutations were seen in 40 different genes among this cohort. Sixty three patients had a single VUS mutation, two patients had two mutations in different genes, whereas six patients had three simultaneous mutations in three different cancer predisposition genes. All different genes with number of mutations in each gene are tabulated in Supplementary Table 3. Interestingly, there were 14 patients (15.7%) with 15 VUS mutations (12.4%) in high penetrance genes (BRCA1-3, BRCA2-4, CDH1-5 and TP53-3) (Table 2). However, among them, only one patient had a significant family history of breast cancer in first-degree relative; however, she is on routine surveillance. A total of 36 (29.8%) VUS mutations were seen in other homologous recombination repair (HRR) pathway genes (ATM-15, RAD50-6, BRCA2-4, BRCA1-3, CHEK2-3, RAD51-2, MRE11-2, ATR-1) (Table 3).

Spectrum of pathogenic mutations seen in 44 patients has been previously reported giving a VUS/pathogenic ratio of 2.02 [12]. The VUS pathogenic ratio for high penetrance genes was 0.38 (13/34). None of the patients with VUS underwent any risk reducing interventions or family testing. No variants were reclassified as pathogenic/likely pathogenic (P/LP) till last follow-up.

## Discussion

In this prospective study on unselected consecutive breast cancer patients at a north Indian tertiary care centre, all of whom underwent multigene hereditary germline panel testing, we found a high rate of VUS mutations (37.71%), although the VUS to pathogenic ratio was low (2.02) owing to a high rate of P/LP mutations in the cohort. More significantly, nearly one third of the mutations were seen in HRR pathway genes (29.8%) with 14 patients (15.7%, BRCA1/2 – 7.9%) having VUS mutations in high penetrance genes further complicating the decision-making process.

Since VUS is defined as a variant with a possibility of association with cancer occurrence between 5.1% and 94.9%, it is apparent that this wide definition accounts for the wide variation in reported prevalence and interpretation. In a large population based study on more than 2,000 women by Tung *et al* [29] across multiple centres in the US, a VUS rate of 41.6% was reported. They tested a relatively young cancer population (median age of 47 years) with 97% having a personal history of breast cancer. This patient disposition was very similar to our study except it was done in a predominantly white population, which likely accounted for a much lower incidence of P/LP mutation and a higher VUS/pathogenic ratio. Similar rates of VUS were reported by Slavin *et al* [30] and Beitsch *et al* [10] in their large datasets from the US using broad multigene panels reflecting consistency of the data. On the other hand, Chong *et al* [31] and Yadav *et al* [9] found lower rates of VUS when a limited panel was used. Our reported rate of 37.7% is much closer to that reported by studies using broad gene panels as our testing panel had 104 genes. It can be argued that using only BRCA1/2 sequencing or limited gene panels could limit the detection of VUS, however, it is unlikely to eliminate it and that would mean missing out on potentially vast amount of genetic information that can be used to inform future decision-making. Moreover, running from the problem is seldom the solution and the more data we collect, the lesser VUS we are likely to find in the future.

There is only one study from India by Singh *et al* [26] that has mentioned VUS rates in their cohort when testing by multigene panel. This was a laboratory-based study on women who were referred for germline testing. Although a 94-gene panel was used, data was reported for 14 high-risk genes and a VUS rate of 15% was found with a 30.1% P/LP mutation rate. A significant referral bias complicates interpretation of this data as VUS rates are generally higher than P/LP rates when using multigene panel testing as seen in large western studies. Our study provides the first prospectively and systematically collected information on VUS from India using a multigene panel and represents the first step in establishing a genetic registry of VUS. We found a VUS/pathogenic ratio of 2.02 which is similar to reported by other large western studies as we tested consecutive unselected patients [9, 10, 29, 30].

Although VUS in any gene complicates decision-making for clinicians and is a source of anxiety for patients, VUS in high penetrance cancer causing genes like BRCA1/2 is especially problematic [32, 33]. Variable VUS rates in BRCA1/2 genes have been reported in various studies ranging from 3.6% in study from US, 6.25% in a study from India, 10.6% from Nigeria and 23.1% from UK (only 52 patients studied) [26, 33–35]. In our cohort, seven patients had a BRCA1/2 VUS (7.9%), similar to that previously seen by Chheda *et al* [27]. Only one patient [36] had a significant family history of breast cancer in her mother and maternal grandmother. Currently, guidelines do not recommend therapeutic decisions based on VUS and these patients should be followed up with routine surveillance [19, 37, 38]. Highlighting the dynamics of complex decision-making in patients with VUS, Welsh *et al* [39] published their experience with 97 patients, all of whom had a BRCA VUS. Interestingly, 22% of these patients underwent a contralateral prophylactic mastectomy (CPM). However, these rates were not different from rates of CPM in other breast cancer patients without a VUS (25%) reflecting patient choice rather than effect of VUS [39]. Similar findings were reported by Chang *et al* [37] who found a VUS in BRCA1/2 in 5.7% of patients but having a VUS did not predict for prophylactic interventions. In our study as well, none of the patients with VUS had any therapeutic interventions in concordance with international guidelines.

However, in the absence of Indian data on genetic mutations, the reliance is on western mutation data which can be problematic. The spectrum and prevalence of P/LP mutations reported from the Indian population is quite different from the west and it stands to reason that same will be the case with VUS [12, 26]. Therefore, establishing a registry of VUS mutations is an urgent need in Indian cancer genetics in order to enable reclassifications based on data generated from a racially and ethnically concordant cohort. This study is a first step in that direction.

In the above-mentioned study by Welsh *et al* [39], 95% of the BRCA VUS were eventually classified as benign; however, 5% were reclassified as P/LP. Slavin *et al* [40] reported that variant reclassification rates can go as high as 17% and can occur as late as 20 years from initial testing, reflecting need for detailed post-test counselling addressing these issues, continued reassessment and regular follow-up in the genetics clinic. As part of mainstreaming genetic testing at our centre, these patients have been under regular follow-up with their primary oncologists with plan to return to genetics clinic if there is reclassification of VUS to P/LP. From our cohort, no VUS has been upgraded till date.

Our study has some strengths and several limitations. This is the first study to provide comprehensive data on spectrum of VUS mutations from unselected Indian breast cancer patients and is likely to add to the growing body of literature on genetic variant information from India. However, a limited number of patients were eventually recruited due to exhaustion of funds and were from a single tertiary care centre which are definite limitations of the study.

## Conclusion

To conclude, VUS remains a significant clinical problem in clinical cancer genetics and this has escalated with expanding access to genetic testing especially with multigene panels. We report a high percentage of VUS (37.7%) in an unselected breast cancer population from India with a VUS pathogenic ratio similar to western studies. This represents the first prospective systematically collected database of VUS mutations in Indian population. This study lays the foundation for further multicentric studies which are currently being planned which will contribute to building up of Indian genetic data registry. Going forward, this will be crucial for reducing the incidence of VUS and therapeutic decision-making.

## Conflicts of interest

All the authors declare no potential conflicts of interest.

## Ethical approval

The study protocol was approved by the IEC vide letter number: IECPG-35/23.01.2019.

## Funding

TATA Trusts at the University of Chicago, New Delhi Center.

## Consent to participate

Informed consent was obtained from all patients.

## Availability of data and material

Data regarding this study will be available from the corresponding author (RP) on reasonable request.

## Authors’ contributions

Authors (RP, OO, AM, SVSD, AG, AB, AK, SB, KSD, ST, and ED) contributed to the concept design and conduct of the study. RP and AM did genetic counselling, AM and RP did the statistical analysis. AM and RP drafted the manuscript and all authors edited the manuscript.

## Figures and Tables

**Supplementary Table 1.** NCCN checklist.

NCCN criteria
An individual with breast cancer diagnosis meeting any of the following: 1. Breast cancer diagnosed < 50 2. Triple negative breast cancer < 60 3. Two breast cancer primaries 4. Breast cancer at any age and: □ >1 close blood relative with ü Breast cancer < 50 or ü Invasive ovarian cancer or ü Male breast cancer or ü Pancreatic cancer or ü High-grade metastatic prostate cancer □ >2 close blood relatives with breast cancer at any age

**Supplementary Table 2.** MCG plus checklist.

MCG plus criteria
An individual with 1. Breast cancer in patient diagnosed ≤ 45 years 2. Two primary breast cancers, both diagnosed in patient ≤ 60 years 3. Triple-negative breast cancer 4. Breast cancer plus parent, sibling or child with any of the above criteria

**Supplementary Table 3.** Details of VUS in all study population.

patient no	gene1	location vus1 (exon)	variantvus1	proteinvus1	gene2	location vus2 (exon)	variant vus2	protien vus2	gene3	location vus3 (exon)	variant vus3	protein vus3
1	TP53	4	c.322G>A	(p.Gly108Ser)								
2	FANCM	22	c.5889del	(p.Val1964TrpfsTer2)								
3	MUTYH	13	c.1231C>T	(p.Gln411Ter)								
4	CDH1	7	c.892G>A	(p.Ala298Thr)								
5	ATM	50	c.7370A>C	(p.Glu2457Ala)								
6	FANCE	2	c.350_351del	(p.Val117AlafsTer11)	CHEK2	11	c.1182G>T	(p.Glu394Asp)				
7	RAD54L	14	c.1511T>C	(p.Ile504Thr)	MSH6	4	c.2489C>G	(p.Ser830Cys)				
8	ATR	9	c.1953del	(p.Trp651Ter)								
9	ATM	14	c.2149C>T	(p.Arg717Trp)								
10	RECQL4	12	c.1925C>G	(p.Ala642Gly)								
11	RECQL4	16	c.2696G>A	(p.Gly899Asp)								
12	BRIP1	3	c.108A>C	(p.Leu36Phe)								
13	RAD54L	11	c.1223T>C	(p.Ile408Thr)								
14	RECQL4	5	c.1073T>G	(p.Met358Arg)								
15	MRE11A	8	c.796C>T	(p.Pro266Ser)	RET	13	c.2290G>A	(p.Ala764Thr)				
16	ATM	31	c.4724G>A	(p.Arg1575His)								
17	MSH3	20	c.2750A>T	(p.Gln917Leu)								
18	CHEK2	12	c.1240C>T	(p.His414Tyr)	RAD54L	11	c.1223T>C	(p.Ile408Thr)				
19	PMS2	14	c.2437C>T	(p.Arg813Trp)								
20	SDHB	4	c.304G>A	(p.Ala102Thr)								
21	BRIP1	10	c.1401A>G	(p.Ile467Met)								
22	FANCF	1	c.484_485del	(p.Leu162AspfsTer103)	ERCC2	20	c.1891C>T	(p.Arg631Cys)				
23	CDH1	2	c.145G>A	(p.Gly49Ser)								
24	RECQL4	4	c.322C>T	(p.Arg108Trp)								
25	ATM	19	c.2873A>G	(p.Glu958Gly)	RAD51C	6	c.873T>G	(p.Asp291Glu)				
26	BARD1	6	c.1559G>A	(p.Arg520Lys)	BRIP1	5	c.445G>T	(p.Asp149Tyr)	NBN	6	c.592C>T	(p.Pro198Ser)
27	FANCE	10	c.1588G>A	(p.Ala530Thr)								
28	RECQL4	5	c.1073T>G	(p.Met358Arg)								
29	SBDS	intron 2	c.258+2T>C	5' splice site								
30	MLH1	6	c.479C>T	(p.Ala160Val)	PMS2	11	c.1211C>G	(p.Pro404Arg)				
31	RAD54L	5	c.320G>A	(p.Arg107Gln)								
32	NBN	10	c.1301C>T	(p.Thr434Ile)								
33	MSH6	6	c.3551T>C	(p.Met1184Thr)	RAD50	11	c.1780C>T	(p.Leu594Phe)	RAD54L	10	c.1093_1169+15dup	
34	RECQL4	11	c.1839G>C	(p.Trp613Cys)								
35	BRCA2	17	c.7884A>G	p.ile2628met								
36	RAD50	19	c.3011T>C	(p.Met1004Thr)								
37	SEC23B	16	c.1754A>G	(p.His585Arg)								
38	BRCA2	5	c.474A>T	(p.Ser158(=))	RECQL4	19	c.3184C>T	(p.Arg1062Trp)				
39	CHEK2	12	c.1240C>T	(p.His414Tyr)	PRF1	2	c.386G>C	(p.Trp129Ser)				
40	ATM	3	c.94C>T	(p.Arg32Cys)	RAD54L	10	c.1093_1169+15dup					
41	RAD50	22	c.3420G>A	(p.Met1140Ile)	FANCI	8	c.655G>T	(p.Val219Phe)				
42	RAD54L	13	c.1460C>T	(p.Ser487Phe)								
43	RAD54L	13	c.1460C>T	(p.Ser487Phe)								
44	MLH3	2	c.911G>T	(p.Gly304Val)								
45	RAD50	16	c.2588T>C	(p.Leu863Pro)								
46	FANCD2	26	c.2433G>C	(p.Lys811Asn)								
47	BRIP1	5	c.493G>A	(p.Glu165Lys)								
48	RAD54L	10	c.1094G>A	(p.Arg365Gln)								
49	MLH3	2	c.716T>G	(p.Ile239Ser)								
50	TP53	5	c.526_528del	(p.Cys176del)	ATM	37	c.5639C>T	(p.Thr1880Met)				
51	WRN	9	c.1161_1163delinsAG	(p.Met387IlefsTer6)								
52	MSH2	intron 8	c.1386+3A>G	(5’ Splice site)								
53	ATM, ATM	27	c.4085G>C	(p.Ser1362Thr)		43	c.6338C>G	(p.Thr2113Ser)				
54	MSH6	9	c.3968T>C	(p.Phe1323Ser)								
55	RAD54L	7	c.575A>G	(p.Gln192Arg)								
56	FANCM	10	c.1706G>A	(p.Arg569His)								
57	MLH1	18	c.2038T>G	(p.Cys680Gly)								
58	FANCM	11	c.1946C>T	(p.Pro649Leu)	PMS2	8	c.880C>T	(p.Arg294Trp)				
59	RAD50	25	c.3858C>G	(p.Phe1286Leu)	RECQL4	16	c.2728G>C	(p.Val910Leu)				
60	BRIP1	12	c.1702A>T	(p.Asn568Tyr)								
61	RAD51D	3	c.212C>T	(p.Ser71Leu)	SDHB	4	c.379A>C	(p.Ile127Leu)				
62	BARD1	4	c.865C>T	(p.Pro289Ser)								
63	BRCA2	25	c.9344A>G	(p.Lys3115Arg)								
64	NF2	1	c.1A>G	(p.Met1?)								
65	MRE11A	4	c.305G>T	(p.Gly102Val)								
66	PMS2	3	c.230A>C	(p.Glu77Ala)								
67	MSH2	1	c.1A>C	(p.Met1?)								
68	FANCE	10	c.1588G>A	(p.Ala530Thr)								
69	ERCC2	6	c.445G>A	(p.Asp149Asn)								
70	TP53	4	c.322G>A	(p.Gly108Ser)								
71	BRCA2	11	c.5357G>A	(p.Ser1786Asn)	RNAsel	4	c.1616T>C	(p.Phe539Ser)				
72	ATM	13	c.2021A>G	(p.His674Arg)	MSH2	3	c.382C>G	(p.Leu128Val)	RECQL4	5	c.803C>T	(p.Pro268Leu)
73	MLH1	intron 14	c.1667+3A>C	(5’ Splice variant)	SEC23B	5	c.539G>T	(p.Cys180Phe)				
74	NBN	2	c.133C>T	(p.His45Tyr)								
75	BRCA1	10	c.2706A>C	(p.Glu902Asp)	CDH1	5	c.592G>A	(p.Asp198Asn)	RAD54L	7	c.605G>A	(p.Arg202His)
76	MSH2	8	c.1386G>A	(p.Gln462(=))								
77	ATM	17	c.2519A>C	(p.Asp840Ala)								
78	BRCA1	22	c.5465G>A	(p.Gly1822Asp)	RAD54L	12	c.1250C>T	(p.Thr417Ile)				
79	ATM	33	c.4997_4999del	(p.Glu1666del)	CDH1	9	c.1223C>T	(p.Ala408Val)	FANCM	2	c.601G>A	(p.Gly201Arg)
80	RECQL4	10	c.1655G>A	(p.Cys552Tyr)								
81	MLH1	11	c.998A>G	(p.Lys333Arg)								
82	NF1	29	c.3972C>T	(p.Thr1324(=))								
83	PMS2	14	c.2396G>A	(p.Arg799Gln)								
84	ATM	37	c.5633C>T	(p.Ser1878Leu)								
85	CDH1	14	c.2234A>G	(p.Glu745Gly)	ATM	3	c.94C>T	(p.Arg32Cys)	ATM	42	c.6163A>G	(p.Ile2055Val)
86	MLH1	8	c.644A>G	(p.Asn215Ser)								
87	BRCA1	10	c.799T>C	(p.Ser267Pro)	ATM	20	c.2980G>A	(p.Val994Ile)				
88	ATM	3	c.94C>T	(p.Arg32Cys)								
89	RAD50	15	c.2425A>G	(p.Ile809Val)								

**Table 1.** Baseline characteristics.

Variable	VUS (*N* = 89)	No VUS (*N* = 147)	*p* value
Age (mean (SD))	44.7 (11.54)	46.2 (11.54)	0.35
Religion (*N* and %) Hindu Muslim Other	77 (86.5) 11 (12.3) 1 (1.1)	132 (89.8) 10 (6.8) 5 (3.4)	0.32
Marital status (*N* and %) Married Unmarried	84 (94.38) 5 (5.6)	141 (95.9) 6 (4.08)	0.59
Clinical stage (*N* and %) I II III IV	5 (5.6) 27 (30.3) 31 (34.8) 26 (29.2)	13 (8.8) 56 (38.1) 50 (34.1) 28 (19)	0.24
ER/PR+ (*N* and %)	40 (44.9)	68 (46.2)	0.84
Her2+ (*N* and %)	38 (42.7)	41 (27.9)	0.019
Triple negative (*N* and %)	27 (30.3)	56 (38.1)	0.22
Significant family history (*N* and %)	7 (8)	27 (22.5)	0.026
NCCN qualifier (*N* and %)	57 (64.4)	108 (73.4)	0.126

ER: estrogen receptor; PR: progesterone receptor

**Table 2.** VUS mutations in high penetrance genes.

High penetrance genes	No of patients	Mutation descriptions
BRCA1	3	exon 10 c.2706A>C (p.Glu902Asp) exon 22 c.5465G>A (p.Gly1822Asp) exon 10 c.799T>C (p.Ser267Pro)
BRCA2	4	exon 17 c.7884A>G p.ile2628met exon 5 c.474A>T (p.Ser158(=)) exon 25 c.9344A>G (p.Lys3115Arg) exon 11 c.5357G>A (p.Ser1786Asn)
P53	3	exon 4 c.322G>A (p.Gly108Ser) exon 5 c.526_528del (p.Cys176del) exon 4 c.322G>A (p.Gly108Ser)
CDH1	5	exon 7 c.892G>A (p.Ala298Thr) exon 2 c.145G>A (p.Gly49Ser) exon 5 c.592G>A (p.Asp198Asn) exon 9 c.1223C>T (p.Ala408Val) exon 14 c.2234A>G (p.Glu745Gly)

**Table 3.** VUS mutations in other HRR pathway genes (except BRCA1/2).

HRR gene	No of patients	Mutation descriptions
ATM	14 (15 mutations)	exon 50 c.7370A>C (p.Glu2457Ala) exon 14 c.2149C>T (p.Arg717Trp) exon 31 c.4724G>A (p.Arg1575His) exon 19 c.2873A>G (p.Glu958Gly) exon 3 c.94C>T (p.Arg32Cys) exon 37 c.5639C>T (p.Thr1880Met) exon 27 c.4085G>C (p.Ser1362Thr) exon 43 c.6338C>G (p.Thr2113Ser) exon 13 c.2021A>G (p.His674Arg) exon 17 c.2519A>C (p.Asp840Ala) exon 33 c.4997_4999del (p.Glu1666del) exon 37 c.5633C>T (p.Ser1878Leu) exon 3 c.94C>T (p.Arg32Cys) exon 20 c.2980G>A (p.Val994Ile) exon 3 c.94C>T (p.Arg32Cys)
RAD50	6	exon 19 c.3011T>C (p.Met1004Thr) exon 22 c.3420G>A (p.Met1140Ile) exon 16 c.2588T>C (p.Leu863Pro) exon 25 c.3858C>G (p.Phe1286Leu) exon 15 c.2425A>G (p.Ile809Val) exon 11 c.1780C>T (p.Leu594Phe)
CHEK2	3	exon 11 c.1223T>C (p.Ile408Thr) exon 2 c.386G>C (p.Trp129Ser) exon 11 c.1182G>T (p.Glu394Asp)
RAD51	2	exon 6 c.873T>G (p.Asp291Glu) exon 3 c.212C>T (p.Ser71Leu)
MRE11	2	exon 8 c.796C>T (p.Pro266Ser) exon 4 c.305G>T (p.Gly102Val)
ATR	1	exon 9 c.1953del (p.Trp651Ter)
